# Hippocampal growth and function are reduced in the newborn following fetal growth restriction

**DOI:** 10.1093/brain/awag061

**Published:** 2026-02-20

**Authors:** Tegan A White, Emily J Camm, Charmaine R Rock, Amy E Sutherland, Yen Pham, Margie Castillo-Melendez, Ingrid Dudink, Elham Ahmadzadeh, Justin Dean, Kirsten R Palmer, Atul Malhotra, Ting Guo, Beth J Allison, Steven P Miller, Suzanne L Miller

**Affiliations:** Department of Obstetrics and Gynaecology, The Ritchie Centre, Monash University, Clayton 3168, Australia; Hudson Institute of Medical Research, Clayton 3168, Australia; Department of Obstetrics and Gynaecology, The Ritchie Centre, Monash University, Clayton 3168, Australia; Hudson Institute of Medical Research, Clayton 3168, Australia; Department of Obstetrics and Gynaecology, The Ritchie Centre, Monash University, Clayton 3168, Australia; Hudson Institute of Medical Research, Clayton 3168, Australia; Department of Obstetrics and Gynaecology, The Ritchie Centre, Monash University, Clayton 3168, Australia; Hudson Institute of Medical Research, Clayton 3168, Australia; Department of Obstetrics and Gynaecology, The Ritchie Centre, Monash University, Clayton 3168, Australia; Hudson Institute of Medical Research, Clayton 3168, Australia; Department of Obstetrics and Gynaecology, The Ritchie Centre, Monash University, Clayton 3168, Australia; Hudson Institute of Medical Research, Clayton 3168, Australia; Department of Obstetrics and Gynaecology, The Ritchie Centre, Monash University, Clayton 3168, Australia; Hudson Institute of Medical Research, Clayton 3168, Australia; Department of Obstetrics and Gynaecology, The Ritchie Centre, Monash University, Clayton 3168, Australia; Hudson Institute of Medical Research, Clayton 3168, Australia; Department of Physiology, Faculty of Health Sciences, The University of Auckland, Auckland 1010, New Zealand; Department of Obstetrics and Gynaecology, The Ritchie Centre, Monash University, Clayton 3168, Australia; Hudson Institute of Medical Research, Clayton 3168, Australia; Women’s Health Research Program, Monash Health, Clayton 3168, Australia; Department of Obstetrics and Gynaecology, The Ritchie Centre, Monash University, Clayton 3168, Australia; Department of Paediatrics, Monash University, Clayton 3168, Australia; Monash Newborn, Monash Children’s Hospital, Clayton 3168, Australia; Neurosciences and Mental Health, The Hospital for Sick Children Research Institute, Toronto, Canada M5G 1X8; Department of Obstetrics and Gynaecology, The Ritchie Centre, Monash University, Clayton 3168, Australia; Hudson Institute of Medical Research, Clayton 3168, Australia; Department of Pediatrics, The Hospital for Sick Children and the University of Toronto, Toronto, Canada M5G 1X8; Department of Pediatrics, BC Children's Hospital Research Institute and University of British Columbia, Vancouver, Canada V5Z 4H4; Department of Obstetrics and Gynaecology, The Ritchie Centre, Monash University, Clayton 3168, Australia; Hudson Institute of Medical Research, Clayton 3168, Australia

**Keywords:** fetal growth restriction, hippocampal development, learning and memory, preclinical and clinical research

## Abstract

The rate of human brain growth is greatest in mid-to-late fetal gestation, corresponding to peak neuronal dendritogenesis. In pregnancies complicated by fetal growth restriction (FGR) caused by placental dysfunction, brain development is adversely impacted, with evidence of reduced total and hippocampal brain volume in childhood and cognitive deficits. The cellular basis for hippocampal maldevelopment and dysfunction in FGR is unknown.

In this study we employed complementary preclinical and clinical investigations of hippocampal developmental trajectory in growth-restricted neonates to address this knowledge gap. In the preclinical study (FGR *n* = 18, control *n* = 19), FGR was induced in fetal sheep via surgical induction of placental insufficiency at 89 days’ gestational age (dGA, term is 148 dGA), and after near-term birth (136 dGA), postnatal memory function was measured over 4 weeks. Neuronal dendritogenesis (dendrite length, branching, complexity) was assessed using Golgi–Cox staining of individual hippocampal cornu ammonis 1 (CA1) neurons at two neonatal time points, newborn age at 24 h after birth and 4 weeks of age, to compare the trajectory of hippocampal neuron development in FGR and control lambs.

Results show that, in control lambs, total dendrite length and branching of CA1 hippocampal neurons significantly increased between newborn age and 4 weeks. In FGR lamb brains, neither dendrite length nor branching increased over this period, and dendrite deficits were worse in FGR lambs at 4 weeks, corresponding to reduced hippocampal area. In 4-week-old lambs, we observed a significant correlation between total dendrite length of CA1 neurons and memory function.

Hippocampal growth trajectory and function deficits were corroborated in small-for-gestational-age (SGA) infants born very preterm. MRI was performed in very preterm infants in early life and repeated at term-equivalent age, showing that the trajectory of anterior hippocampal growth was significantly reduced in SGA infants (*n* = 20) compared to appropriate-for-gestational age (*n* = 139) infants. Within the SGA infant cohort, reduced anterior hippocampal volume was significantly associated with lower Bayley Cognitive Composite scores at 18 months corrected age, controlling for gestational age at birth and infant sex. Together, these findings demonstrate that the trajectory of hippocampal growth is significantly impaired in SGA/FGR infants, caused by disturbed neuronal dendritogenesis that is programmed by an adverse fetal environment and persists, or worsens, after birth. Results in the clinical cohort provide the critical link between programming of reduced dendritogenesis, hippocampal volume deficit and cognitive dysfunction in SGA infants.

## Teaser

White *et al.* combine human and animal data to show that fetal growth restriction impairs postnatal brain development. Low birth weight is associated with reduced dendritic outgrowth in hippocampal neurons, suggesting that impaired dendritogenesis is programmed *in utero* and leads to lasting structural and functional deficits.

## Introduction

The final trimester of human pregnancy from ∼28 weeks’ gestation onwards corresponds to the peak trajectory in fetal brain growth, particularly for increasing grey matter volume.^1^ Rapid grey matter expansion is underpinned by neuronal dendritogenesis, synaptogenesis, myelination and gliogenesis.^2^ Preterm birth and fetal antenatal compromise, such as with fetal growth restriction (FGR), disrupt these foundational neurodevelopmental processes. FGR causes lifelong deficits in motor function, cognition and behaviour.^3,4^ In human infants born very preterm at <32 weeks’ gestation and/or FGR, aberrant brain development can be detected as reduced total brain volume, or grey matter volume, at term-equivalent age or in early infancy.^5-7^

FGR describes a pathological disruption to fetal growth, principally caused by impaired oxygen and nutrient transfer secondary to placental insufficiency.^8,9^ In FGR fetuses, chronic fetal hypoxia adversely impacts development of multiple organ systems, including the brain.^8,10^ Historically, the terms small for gestational age (SGA) and FGR were used interchangeably, with both associated with neurodevelopmental impairments.^11,12^ Since 2016, the consensus definition of FGR describes the condition as an estimated fetal weight less than the 10th percentile for gestation and sex, together with antenatal Doppler parameters of placental dysfunction (umbilical artery and/or uterine artery) to confirm placental insufficiency.^13^ In the absence of antenatal Dopplers, SGA is used as a proxy for high-risk small infants.^14^ In SGA/FGR infants, suboptimal brain development is first evident *in utero*, with MRI revealing reduced brain volume.^15,16^ After birth, total brain volume is reduced in growth-restricted infants, with vulnerability in neuron-rich cortical and hippocampal grey matter.^5,17^

The hippocampus is critical for acquiring, processing and accessing memories, and emotional regulation.^18^ The hippocampus is populated by pyramidal neurons, with hippocampal neurogenesis mostly complete by mid-gestation, followed by rapid volume expansion.^19^ Volume increase is driven by apical and basal dendrite outgrowth to produce large and complex neuronal arbours.^20,21^ In FGR infants born preterm who underwent 3D MRI scan at term-equivalent age, Lodygensky and colleagues showed that hippocampal volume was reduced and correlated with poor outcomes on neonatal neurobehavioural assessments.^17^ At school age, deficits in learning and memory are evident in children born FGR/SGA.^17,18,22-24^ A study in growth-restricted sheep showed reduced trajectory of hippocampal dendritogenesis in the fetal brain,^25^ but whether aberrant dendritogenesis and reduced hippocampal arborization persist after birth, cause volume loss or are associated with functional learning deficits is unknown.

The current study employed complementary preclinical and clinical cohorts, including a well-characterized sheep model of FGR caused by placental insufficiency,^26^ induced at 89 days’ gestational age (dGA, 0.6 gestation), together with human very preterm infants stratified for SGA or appropriate for gestational age (AGA) with serial neonatal brain MRI. In both the clinical and preclinical studies, two neonatal time-point measures of hippocampal structure were assessed, allowing quantification of hippocampal growth trajectory. Functional testing was undertaken in clinical and preclinical cohorts, with functional indices of cognition related to hippocampal size. The sheep study permitted cellular-level examination of neuron morphology and dendritogenesis in near-term control and FGR lambs at 24 h after birth (newborn) and 4 weeks of age. These corresponding preclinical and clinical data are the first to detail the adverse effects of FGR on the trajectory of hippocampal growth contributed by a sustained adverse disruption of neuronal microstructure, hippocampal volume and subsequent impact on function. The results provide translational insight into conserved disruption to hippocampal development, with cellular level evidence to underpin structural vulnerability, and informs the clinical trajectory of neurodevelopmental impairment following impaired fetal growth.

## Materials and methods

### Preclinical sheep experiments

Experimental procedures were approved by Monash Medical Centre A animal ethics (MMCA2016/19, 2019/02), complying with NHMRC Australia Code of Practice for the Care and Use of Animals for Scientific Purposes and ARRIVE guidelines.

#### Fetal surgery and experimental procedures

Surgery was performed on 37 singleton-bearing pregnant sheep at 89 dGA (term 148 dGA). Ewes were randomized to control (*n* = 19) or FGR (*n* = 18) at surgery, with single umbilical artery ligation used to induce early-onset FGR.^26,27^ Preterm birth was induced with 50 mg mifepristone (Mifepristone Linepharma, MS Health) at 134 dGA and 11.4 mg betamethasone (Celestone Chronodose, Merck) at 134 and 135 dGA, with all lambs born near-term at 136 dGA. Two age cohorts were assessed, newborn lambs at 24 h, and 4-week-old lambs. All lambs were monitored and weighed daily. The 4-week-old lambs performed behavioural assessments from 2 to 4 weeks of age as described below. Lambs were euthanized at their respective newborn and 4-week time points (100 mg/kg IV, Lethobarb, Virbac), and plasma samples and brains collected.^26,28^

#### Lamb behavioural testing

Lamb cognitive tests followed Camm *et al.*,^29^ and used the T-maze between 2 and 4 weeks of age. The T-maze assesses spatial navigation, memory and recognition and cognitive flexibility of the lambs. The T-maze uses two ewes positioned in cages at each T end of the maze, one being the lamb’s mother and the other a non-related ewe. This task was performed three times each week and the location of the ewes within the left or right arms of the T-maze was randomly determined before each trial, with at least one switch in positioning over the three trials. Time taken to complete the maze and errors made were assessed. Errors included going to the wrong ewe, going back and forth in the maze or returning to the starting position.

#### Biomarker analyses

In neonatal plasma we measured malondialdehyde (MDA) using a TBARS kit (Cayman Chemical); the assay range was 0–50 µM, 13% inter-assay and 6% intra-assay coefficients of variation. Cytokine and chemokine concentrations were quantified in plasma with the Luminex® xMAP® (Luminex Corporation,) and a MILLIPLEX® panel. We measured interleukin 6 (IL-6), IL-10 and tumour necrosis factor α (TNF-α) (Merck KGaA), all with <10% intra-assay and <15% inter-assay coefficients of variation, and accuracy >92%.

#### Hippocampal cornu ammonis 1 neuronal morphology and immunohistochemistry

The brain was removed, weighed and left hemisphere hippocampal slices were processed for Golgi–Cox staining using the Rapid Golgi technique (FD NeuroTechnologies, Cell Systems Biology) and right hemisphere slices processed for histology and immunohistochemistry, as described.^28,30,31^

#### Hippocampal width and cellular analysis

In the ventral hippocampus, total area and cornu ammonis 1 (CA1) sublayer widths were quantified on cresyl violet (0.5%) and acid fuchsin (1%) stained slides, and immunostaining of neurons (NeuN), microglia (ionized calcium binding adaptor molecule 1, Iba-1), astrocytes (glial fibrillary acidic protein, GFAP), neuronal cytoskeletal structure (microtubule-associated protein 2, MAP2) and synapsin (Syp-1) were performed on paraffin-embedded sections (Supplementary Table 1). Images of the hippocampus were taken at ×40 under light or fluorescence microscopy (Olympus BX41 DP25). Area and width analysis was conducted on two slides per animal, with point counting for area and CA1 width calculated from 10 averaged measurements.^32^ ImageJ was used to manually quantify cell density (NeuN) across two slides per animal. Microglial and astrocyte quantification was performed on six CA1 fields of view per animal, using Iba-1- and GFAP-stained slides visualized with DAB (3,3′-diaminobenzidine). Microglia were manually examined and quantified based on physical characteristics to describe shape as amoeboid, intermediate or resting phenotype,^33^ and astrocytes were manually quantified as either reactive or resting phenotype.^34^ Fluorescent double-labelling was performed on Iba-1 with MAP2 to visualize cell-to-cell relationships. Immunohistochemical analysis of MAP2 and Syp-1 was conducted with an ImageJ automated density quantification macro with thresholding consistent across each stain for six CA1 fields of view per animal. Golgi–Cox-stained CA1 pyramidal neurons were imaged, and 179 pyramidal neurons (6–8 neurons per animal) were selected and analysed as previously described.^28^ One investigator (T.A.W.) analysed coded slides.

#### Lamb statistical analysis

Data were assessed for normality using Shapiro–Wilk test and are expressed as mean ± standard deviation (SD), and significance was accepted at *P* < 0.05 using Prism (v.10.6.1, GraphPad Software, USA). Statistical comparisons of FGR and control lambs were performed using two-way ANOVA followed by Tukey’s *post hoc* test (or multiple Mann–Whitney U-tests for data not normally distributed). Simple linear regression was performed on cumulative T-maze errors to total ventral hippocampal area.

### Human study

#### Human study participants

This prospective cohort study enrolled 166 very preterm infants (24–32 weeks’ gestation) with detailed clinical data and neonatal brain MRI between 2013 and 2019 at The Hospital for Sick Children (SickKids) and Mount Sinai Hospital, both tertiary-level neonatal intensive care units (NICU) in Toronto, Canada. Within this very preterm cohort, 20 SGA, 7 large for gestational age (LGA) and 139 AGA infants were assessed. The Research Ethics Boards of both hospitals reviewed and approved this study. Written informed consent was obtained from the parents/guardians. Pregnancy, delivery and neonatal course data were collected through daily chart review. SGA and LGA were defined as below 10th and above 90th percentile for birth weight, respectively.^35^ Given the preclinical data focused on FGR and differences in the pattern of comorbidities between AGA and LGA infants, SGA infants were only compared with AGA infants and LGA infants were excluded in subsequent analyses.

#### Human MRI

Very preterm infants underwent early-life MRI scans as soon as they were clinically stable and again at term-equivalent age, as detailed previously.^24^ Segmentations of the left and right hippocampi of each infant were performed on the native 3D T1-weighted image at early-life and/or at term-equivalent age using a well-established method.^24,36^ The mean hippocampal volume of both hemispheres on each scan was calculated for each preterm infant. The hippocampus was further divided into anterior and posterior subregions, using the uncal apex of the parahippocampal gyrus as the landmark.^24^ For statistical analyses, the mean volume of left and right sides for the anterior hippocampal region and that of the posterior hippocampal region for each preterm infant at each time point were included.

#### Human neurodevelopmental outcomes

Cognitive outcome at 18 months’ corrected age were assessed by experienced personnel at the Neonatal Follow-up Program using the Bayley Scales of Infant and Toddler Development (Bayley-III).^24^ Bayley Cognitive Composite scores with mean of 100 and standard deviation of 15 were used in statistical analyses.

#### Human statistical analysis

R (v.4.4.2) and R Studio (v.2025.09.2+418) were used for statistical analysis. Clinical characteristics and demographic variables between birth weight groups were compared using chi-square or Fisher’s exact test for categorical data and Mann–Whitney test for continuous data. Hippocampal volumes of both hemispheres were not significantly different; thus, the means of bilateral hippocampal volumes were used in subsequent statistical analyses. To account for repeated measures, generalized estimating equation (GEE) models were developed using an independent correlation structure and robust standard errors correction. Associations between neonatal anterior and posterior hippocampal volumes with birthweight group, post-menstrual age (PMA) at MRI and their interaction term were assessed in separate models. Associations between cognitive outcome and the percentages of anterior and posterior hippocampal volumes relative to total cerebral volume with an interaction term of birth weight group were also analysed with GEE. To take into account key covariates for cognitive development in preterm infants, additional models were adjusted for each of the clinical factors, including birth GA, PMA at MRI, sex, as well as those that are significantly different between SGA and AGA very preterm infants respectively and together.

## Results

### Fetal growth restriction

All lambs were born near-term (136 dGA) and FGR lambs weighed ∼29% less than control lambs at birth (*P* < 0.001; Table 1). All lambs in the 4-week cohorts gained weight postnatally (*P* < 0.001); however, the FGR lambs remained lighter compared to controls (*P* < 0.05). FGR lambs demonstrated asymmetric growth restriction at both newborn and at 4 weeks of age, with an increased brain-to-body weight ratio (*P* < 0.05).

**Table 1:** Lamb weights, biometry

	Newborn		4-week	
	Control	FGR	Control	FGR
Number of animals	*n* = 8	*n* = 9	*n* = 11	*n* = 9
Sex	5 females, 3 males	5 females, 4 males	4 females, 7 males	4 females, 5 males
Birth weight (kg)	4.6 ± 0.9	**3.1** ± 0.9*	4.4 ± 0.4	**3.2** ± 0.2*
PM weight (kg)	5.0 ± 0.3	**3.2 ± 0.3** *	13.3 ± 1.3	**11.0 ± 1.5** *
Brain weight (g)	53.4 ± 2.9	**49.0 ± 4.0** *	73.5 ± 4.2	71.1 ± 4.9
Brain/body weight (g/kg)	11.0 ± 2.4	**16.5 ± 5.2** *	5.5 ± 0.5	**6.5 ± 0.8** *
CRL (cm)	49.8 ± 6.7	**41.9 ± 6.9** *	72.2 ± 4.3	**62.7 ± 4.5** *
BPD (cm)	7.0 ± 0.8	**6.0 ± 0.7** *	9.2 ± 0.6	8.6 ± 1.0
Plasma MDA concentration (µM)	10.2 ± 2.0	**14.5 ± 2.1** *	8.7 ± 3.4	7.9 ± 2.4
Plasma IL-6 (pg/ml)	239.3 ± 56.2	**771.3 ± 197.4** *	394.3 ± 563.9	329.3 ± 234.9
Plasma IL-10 (pg/ml)	867.3 ± 165.4	782.1 ± 213.7	508.8 ± 393.0	382.9 ± 414.5
Plasma TNF-α (pg/ml)	1196 ± 274.7	1200 ± 397.3	411.5 ± 392.6	714.5 ± 417.6

Data are mean ± standard deviation. Normality was assessed for all groups. Statistical comparisons of FGR and control lambs were made using a two-way ANOVA followed by Tukey’s *post hoc* analysis where interactions were observed. When data were not normally distributed, multiple Mann–Whitney U-tests were performed. Significantly different values are indicated in bold text. BPD = biparietal distance; CRL = crown rump length; FGR = fetal growth restriction; IL-6 = interleukin 6; IL-10 = interleukin 10; MDA = malondialdehyde; PM = post-mortem; TNF-α = tumor necrosis factor-α.

^*^ *P* < 0.05 versus age-matched control.

### Cognitive deficits in FGR lambs

To assess learning and memory function of our 4-week control and FGR cohorts, we used the T-maze task across three consecutive postnatal weeks. FGR lambs demonstrated significant spatial navigation and memory deficits within the T-maze compared to control lambs, where FGR lambs had increased cumulative errors overall (*P* = 0.016; Fig. 1B) and increased errors across all testing weeks (*P* = 0.037; Fig. 1C). There was no group difference in the time taken to complete the maze (Fig. 1D). Greater number of errors in the FGR lambs without a corresponding increase in completion time indicates a potential difference in cognitive strategy, impaired behavioural regulation or heightened impulsivity during task performance.

**Figure 1:**
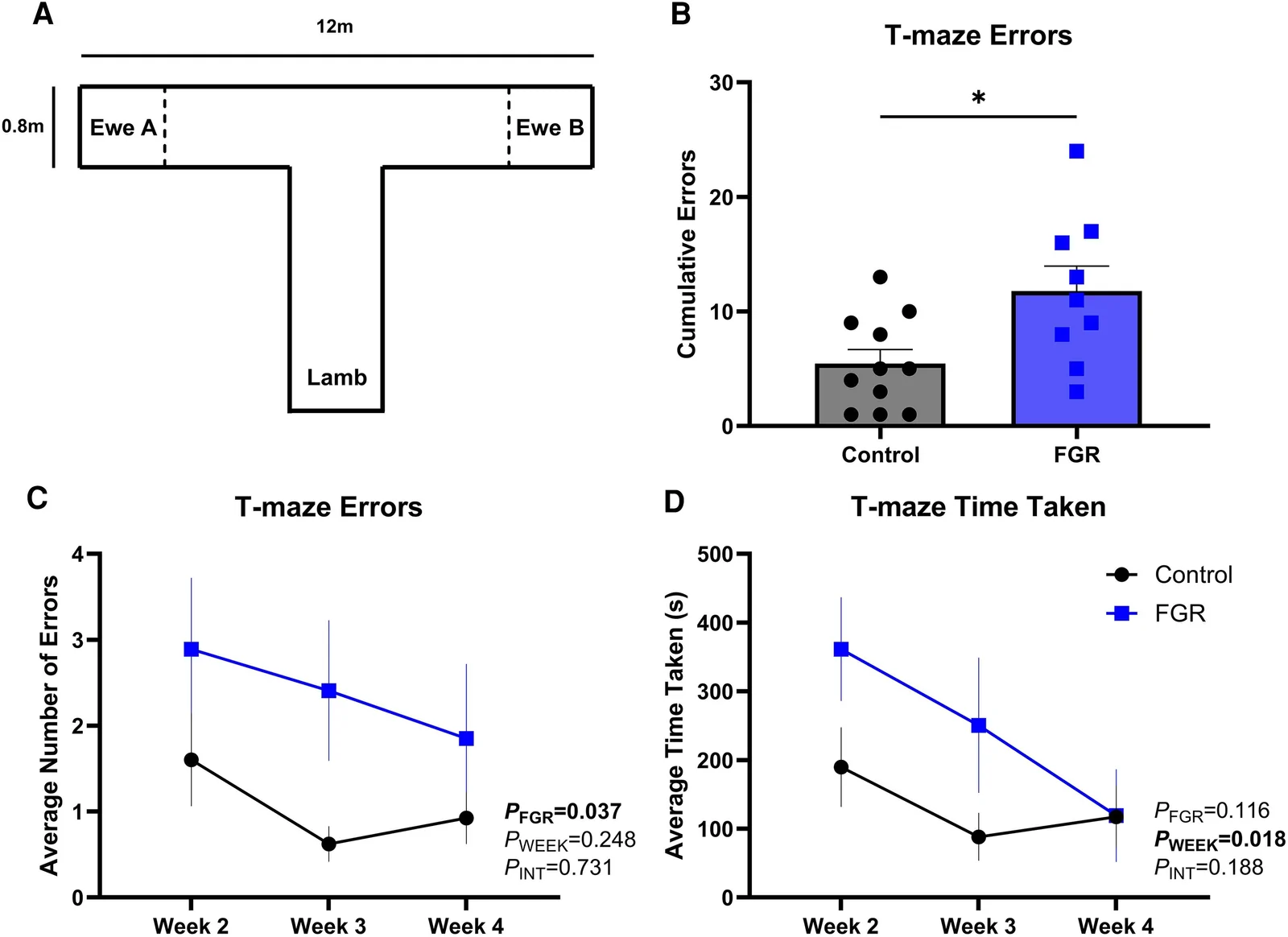
**Four-week lamb cognitive assessment, T-maze.** (**A**) Diagram of the T-maze. (**B**) Cumulative errors performed across all weeks of testing; (**C**) average number of errors performed across each testing week; and (**D**) average time taken to complete the maze in each testing week. Data are mean ± standard deviation. *P < 0.05. Unpaired students *t*-test for graph in **B**. Two-way ANOVA for graphs in **C** and **D**, with group and test week as variables. Tukey’s multiple comparisons *post hoc* test. Significant results are in bold text. FGR = fetal growth restriction.

### Plasma oxidative stress and inflammation are increased in newborn FGR lambs

In newborn FGR lambs, plasma MDA (*P* = 0.002) and IL-6 concentrations (*P* = 0.027) were increased relative to newborn control lambs, demonstrating increased systemic oxidative stress and inflammation (Table 1). At 4 weeks of age, no indices of circulating oxidative stress or inflammation were evident in either cohort.

### FGR disrupts hippocampal morphology

Progressive expansion in total hippocampal area and widths of the individual stratum oriens (SO) and stratum radiatum and stratum lacunosum-moleculare (SR and SL-M) CA1 layers combined were observed in the hippocampus of control lambs between the newborn and 4-week time points (Fig. 2C and D). Conversely, FGR offspring showed an increase in hippocampal area (*P* < 0.001) and SO layer width (*P* = 0.003) between newborn and 4-week time points, but with no increase observed in SR and SL-M layer width in which the apical dendritic arbours are located (*P* = 0.218; Fig. 2C). The width of the CA1 hippocampal SR and SL-M layer was significantly reduced in newborn FGR lambs compared to control lambs (*P* = 0.046; Fig. 2C). In the 4-week FGR cohort, hippocampal area was reduced in the FGR group relative to controls (Fig. 2D), with a concomitant reduction in SR and SL-M layer widths (*P* = 0.002; Fig. 2C). We determined whether aberrant hippocampal growth in FGR lambs was related to loss of pyramidal neurons (Fig. 2). Neuronal density within the stratum pyramidale (SP) layer did not change with age (*P* = 0.082) or FGR (*P* = 0.533; Fig. 2B).

**Figure 2:**
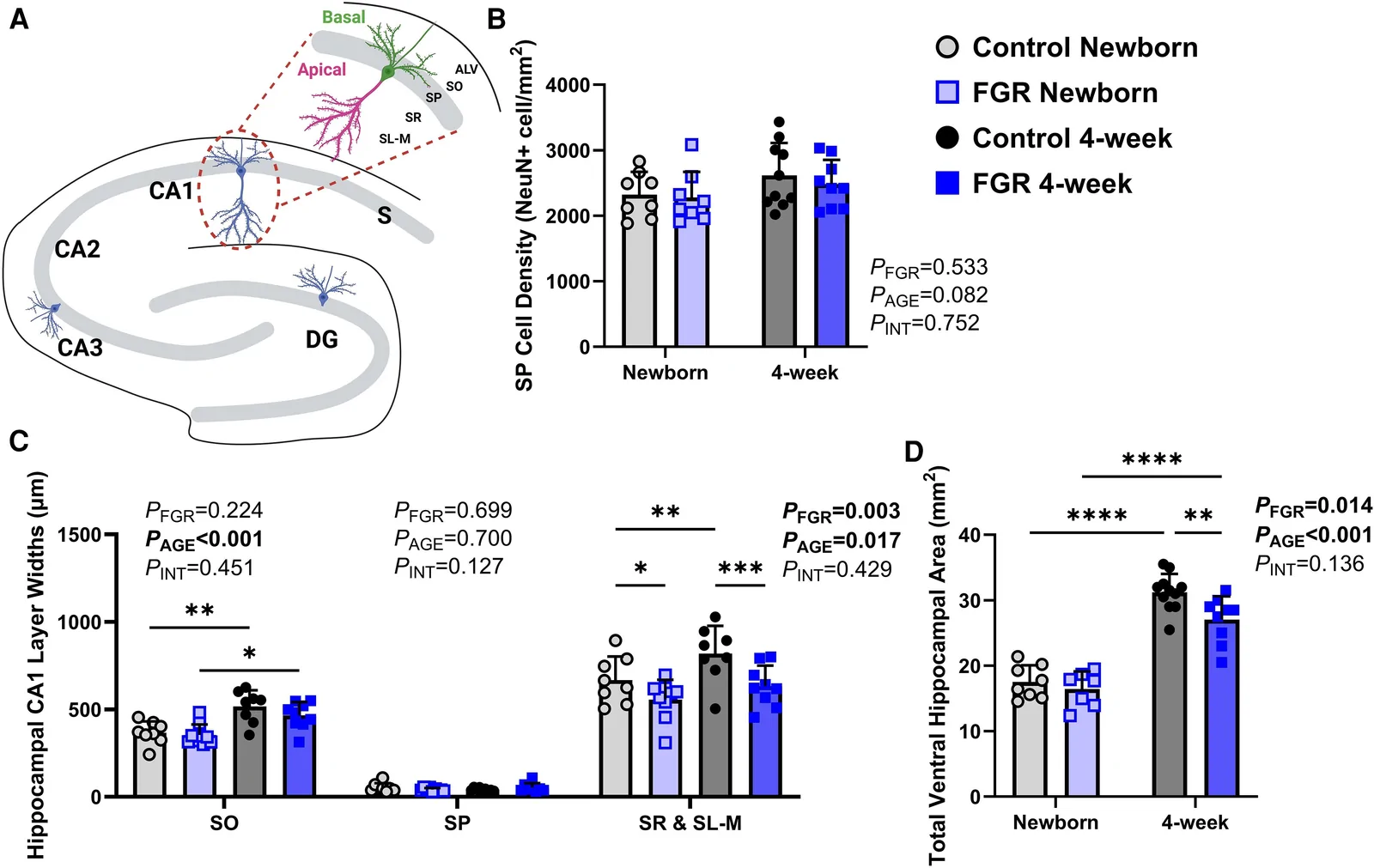
**Hippocampal morphology.** (**A**) Representative image of the ovine hippocampus and a CA1 pyramidal neuron. (**B**) Analysis of NeuN-positive cell density. (**C**) Width of CA1 layers. (**D**) Analysis of hippocampal area. Data are mean ± standard deviation. Two-way ANOVA for graphs in **B**, **C** and **D**, with age and group as variables; Tukey’s multiple comparisons *post hoc* test. *P < 0.05, **P < 0.01, ***P < 0.001, ****P < 0.0001. Significant results are in bold text. Open symbols indicate newborn cohorts; filled symbols indicate 4-week cohorts. CA = cornu ammonis; FGR = fetal growth restriction; SO = stratum oriens; SP = stratum pyramidale; SR = stratum radiatum; SL-M = stratum lacunosum-moleculare. Figure partially created in BioRender. White, T. (2026) https://BioRender.com/r9dkhzj

### Neuronal morphology and complexity impairments in FGR lambs

We next postulated that disrupted growth and expansion of the hippocampus in FGR offspring would be accompanied by abnormalities in neuronal microstructure, evaluated using Golgi–Cox staining (Fig. 3). In control lambs, summated (apical plus basal) dendrite length (*P* < 0.001; Fig. 3B) and branching (*P* = 0.039; Fig. 3C) of CA1 neurons increased between newborn age and 4 weeks, but not in FGR lambs (*P* = 0.068 and *P* = 0.264, respectively). In newborn FGR lambs, summated branch points were reduced (*P* = 0.031; Fig. 3C) but not summated dendritic length (*P* = 0.144; Fig. 3B). At 4 weeks, summated branch points (*P* = 0.001; Fig. 3C) and summated dendritic length (*P* = 0.001; Fig. 3B) were both reduced in FGR lambs. At the newborn age, the number of basal branch points was reduced in FGR lambs compared to controls (*P* = 0.008; Fig. 3D), but total basal dendrite length and complexity were unaffected. In apical dendrites, both branching (*P* < 0.005; Fig. 3E) and length (*P* = 0.040; Fig. 3G) were reduced in newborn FGR offspring. Having confirmed that FGR was associated with altered hippocampal neuronal cytoarchitecture at birth, we examined whether neuropathology persisted or was resolved at 4 weeks. FGR offspring showed a persistent deficit in apical branch points (*P* = 0.002; Fig. 3E), apical length (*P* < 0.001; Fig. 3G) and apical complexity (*P* = 0.009; Fig. 3I) at 4 weeks. Basal dendrites showed an overall deficit in branching in FGR offspring (Fig. 3D).

**Figure 3:**
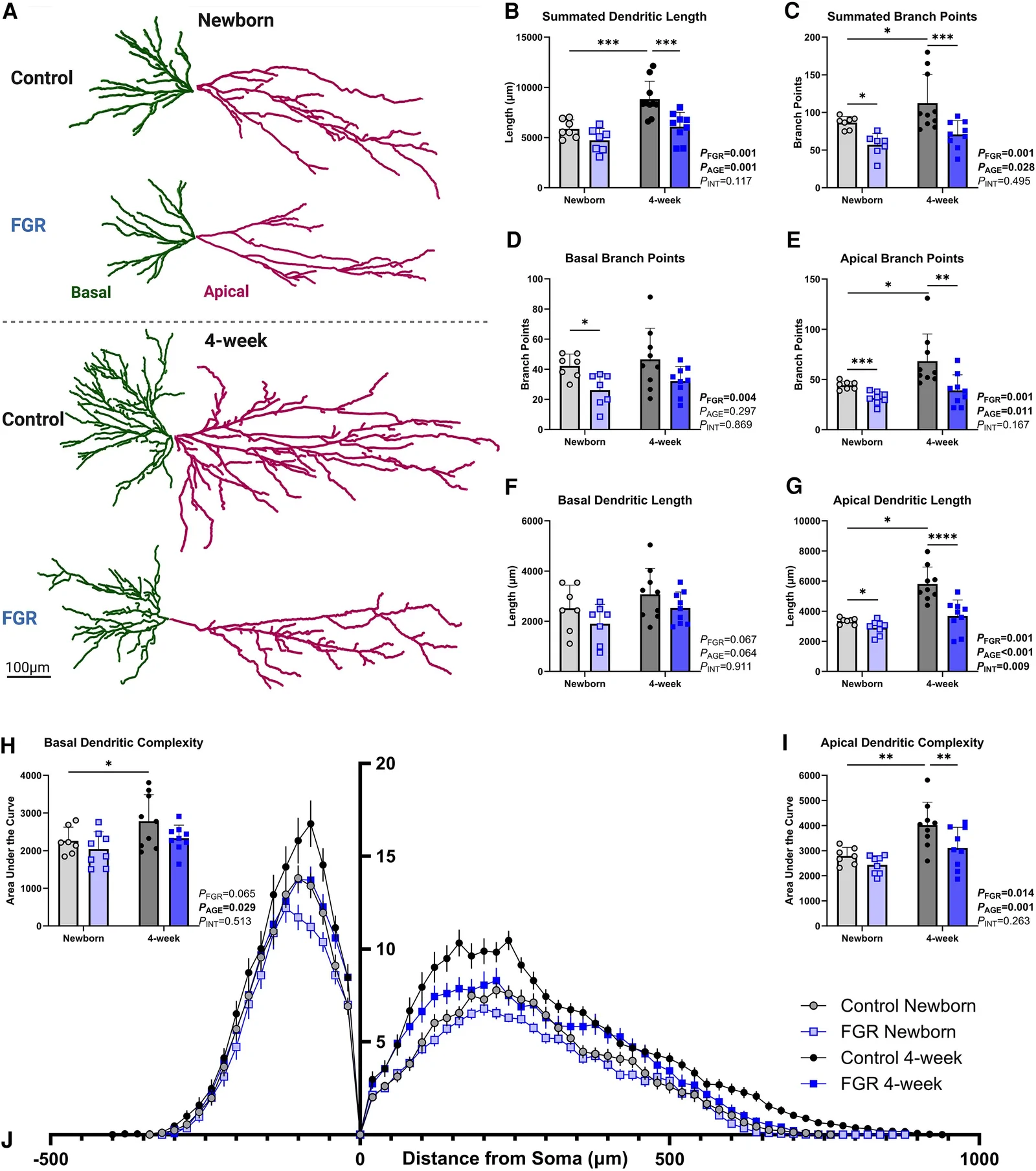
**Hippocampal neuronal analysis using Golgi-stained neurons.** (**A**) Representative average tracings of newborn and 4-week hippocampal neurons of control and fetal growth-restricted (FGR) lambs. (**B**) Summated dendritic length. (**C**) Summated branch points. (**D** and **E**) Average number of branch points, basal (**D**) and apical (**E**). (**F** and **G**) Average dendritic length, basal (**F**) and apical (**G**). (**H** and **I**) Analysis of dendritic complexity with area under the curve of the Sholl analysis for basal dendrites (**H**) and apical dendrites (**I**). (**J**) Sholl analysis. Data are mean ± standard deviation. Two-way ANOVA with age and group; Tukey’s *post hoc* test. *P < 0.05 **P < 0.01, ***P < 0.001, ****P < 0.0001. Significant results are in bold text. Scale bar = 100 µm.

In control lambs, there was a significant increase in the trajectory of neurite outgrowth for measures of basal arbour complexity (*P* = 0.029; Fig. 3H), apical branch number (*P* = 0.036; Fig. 3E), apical dendrite length (*P* < 0.001; Fig. 3G), apical arbour complexity (*P* < 0.001; Fig. 3I), summated branch points (*P* = 0.040; Fig. 3C) and summated dendritic length (*P* = 0.001; Fig. 3B) from newborn to 4 weeks. This growth trajectory from newborn to 4 weeks was not observed in the FGR cohorts for any neuronal microstructure outcome.

Reduction in dendritic arborization, predominately observed in apical dendrites, was confirmed by immunohistochemical staining with MAP2, a cytoskeletal neuronal marker, which was found to be decreased in FGR offspring in SR and SL-M layers at birth (*P* = 0.006; Fig. 4E) persisting at 4 weeks (*P* = 0.029; Fig. 4E). There was an overall deficit in synapsin density in the SO layer in FGR offspring (*P* = 0.007; Fig. 4C).

**Figure 4:**
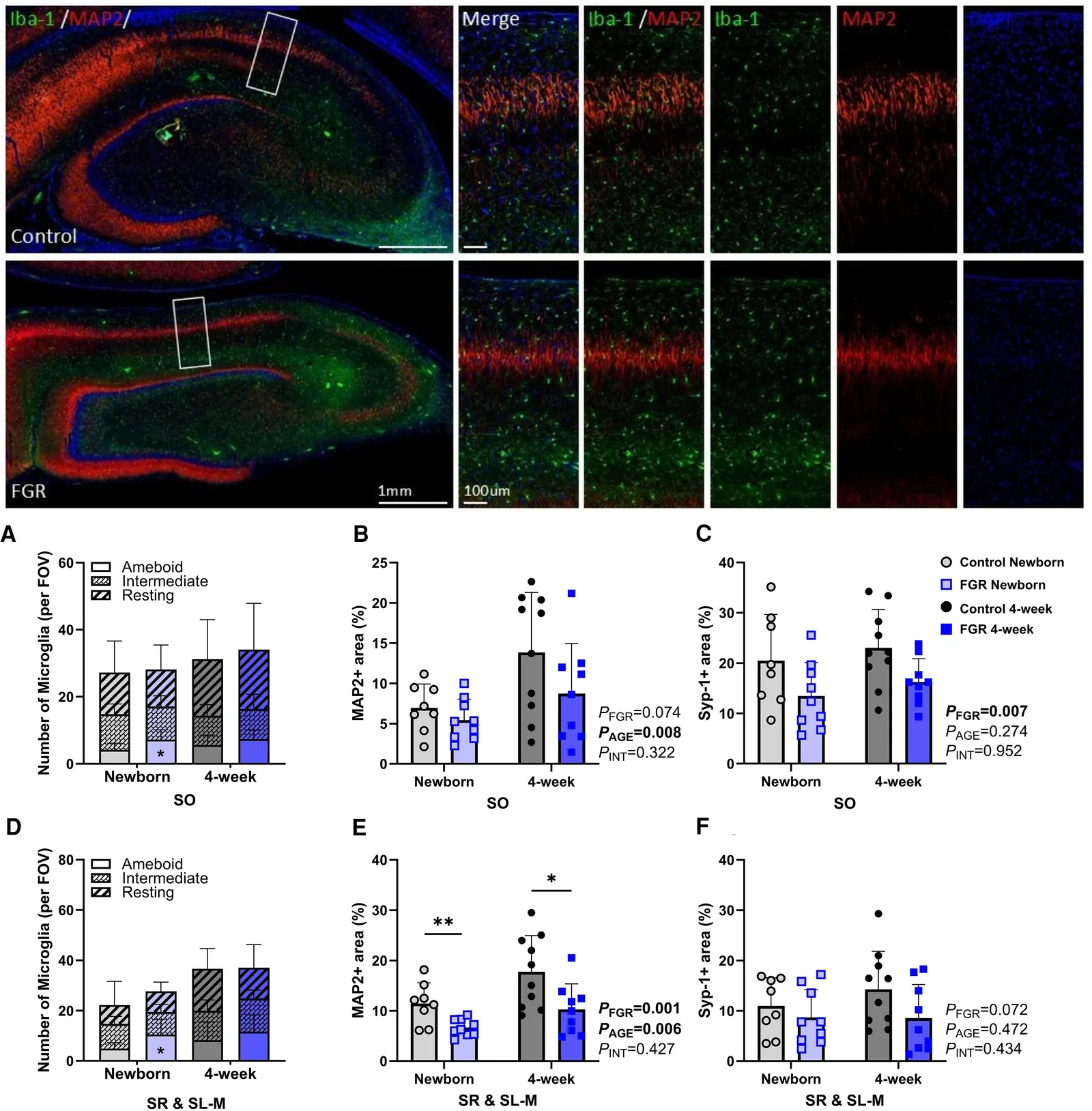
**Hippocampal immunohistochemical analysis.**  *Top*: Representative images of microtubule-associated protein 2 (MAP2) and ionized calcium-binding adaptor molecule 1 (Iba-1) staining in newborn control and fetal growth-restricted (FGR) lambs. (**A**) Iba-1-positive cells quantified in morphological subtypes of amoeboid, intermediate and resting, in stratum oriens (SO) and (**D**) stratum radiatum and stratum lacunosum-moleculare (SR & SL-M) layers. (**B**) Percentage area of positive MAP2 staining in SO and (**E**) SR & SL-M layers. (**C**) Percentage area of positive synapsin 1 (Syp-1) staining in SO and (**F**) SR & SL-M. Data are mean ± standard deviation. Two-way ANOVA with age and group; Tukey’s *post hoc* test. *P < 0.05, **P < 0.01. Significant results are in bold text. Scale bar =1 mm and 100 µm. FOV = field of view.

A significant, albeit subtle correlation was observed between the summated number of branch points and total hippocampal area (*P* = 0.046, *r*^2^ = 0.214) and summated dendritic length and total hippocampal area (*P* = 0.038, *r*^2^ = 0.229; data not shown).

### Increased neuroinflammation in FGR lambs

Immunohistochemical staining for Iba-1-positive microglia showed no difference in the density of microglia between the control and FGR groups in either SO or SR and SL-M layers. However, the FGR cohort showed an increase in the amoeboid phenotype of microglia at birth, indicative of a proinflammatory microglial state. This phenotype was evident in both the SO (*P* = 0.041; Fig. 4A) and combined SR and SL-M layers relative to control (*P* = 0.049; Fig. 4D). There were no observed differences in microglial density or activation state between control and FGR at 4 weeks. Astrocyte morphology and density were evaluated using GFAP immunohistochemical staining and there were no significant differences in astrocyte density between control and FGR groups within the SO or SR and SL-M layers (Supplementary Fig. 1). Further, there were no detectable differences in the proportion of astrocytes exhibiting activated or resting phenotypes between the groups (Supplementary Fig. 1).

### Hippocampal structural abnormalities associated with cognitive deficits in FGR lambs

Simple linear regressions were performed to determine if a structure to function relationship exists within the outcomes included in this study (Fig. 5). There was no significant correlation between total ventral hippocampal area and cumulative T-maze errors in the 4-week lamb cohorts (*P* = 0.098, *r*^2^ = 0.152; Fig. 5A); however, a significant negative correlation was observed between summated dendrite length and cumulative T-maze errors (*P* = 0.024, *r*^2^ = 0.267; Fig. 5B).

**Figure 5:**
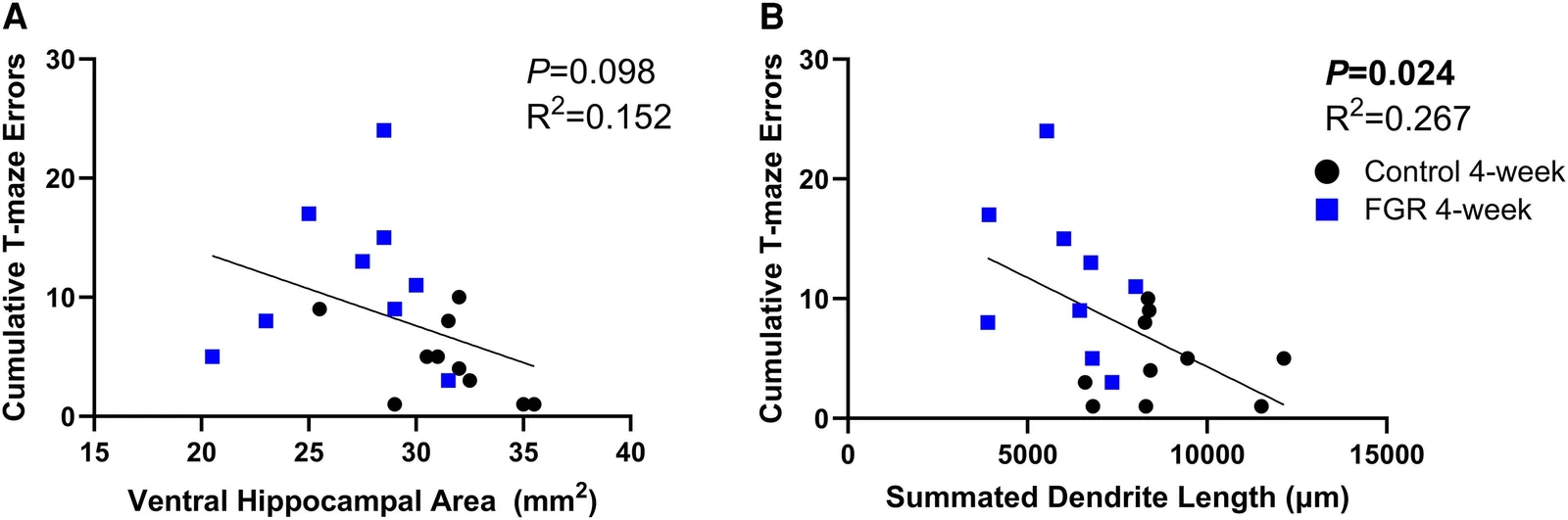
**Structure to function correlation.** (**A**) Simple linear regression of ventral hippocampal area versus cumulative T-maze errors. (**B**) Simple linear regression of summated dendrite length and cumulative T-maze errors. Significant results are in bold text.. Black symbols = control, blue symbols = fetal growth restriction (FGR).

### Reduced hippocampal development in SGA human very preterm infants

To explore the clinical relevance of findings from the FGR sheep model, we next examined hippocampal development in human very preterm infants diagnosed as SGA (Table 2), as it is frequently used as a surrogate marker of FGR in preterm infants. Among the clinical factors, birth GA (*P* = 0.02), Apgar score less than 8 at 5 min (*P* = 0.03), days of mechanical ventilation (*P* = 0.004) and postnatal corticosteroids (*P* = 0.04) were significantly different between SGA and AGA infants. Hippocampal volume in human preterm SGA and AGA infants was plotted against PMA at MRI from early life to term-equivalent age (Fig. 6). A difference in growth trajectories of the anterior hippocampus from early life to term-equivalent age was observed between the SGA group and the AGA group (Fig. 6B). GEE models examined the association of birthweight group and PMA at MRI on total, anterior or posterior hippocampal volume, while also considering the interaction of birthweight group and PMA at MRI. The results of the GEE model, where the interaction term was significantly associated with the anterior hippocampal volume (β = 3.494, *P* = 0.048), demonstrated that anterior hippocampal volume from early life to term-equivalent age in AGA group increased more robustly with the increase of PMA at MRI compared to the SGA group. To account for total cerebral volume, the percentages of anterior, posterior and total hippocampal volumes relative to total cerebral volume were used in another set of GEE models. The interaction of anterior hippocampus percentage and SGA group was significantly associated with higher Bayley Cognitive Composite scores at 18 months (interaction term *P* = 0.020) in the basic model. When controlling for each of the key clinical factors respectively, Bayley Cognitive Composite scores were associated with gestational age at birth (β = 1.287, *P* = 0.001), postnatal corticosteroids (β = −5.82, *P* = 0.009) and total days of mechanical ventilation (β = −0.134, *P* = 0.002), but not with sex, PMA at MRI and Apgar score <8 at 5 min. The association between cognitive outcome and the interaction of anterior hippocampus percentage and SGA group remained significant in all models.

**Figure 6:**
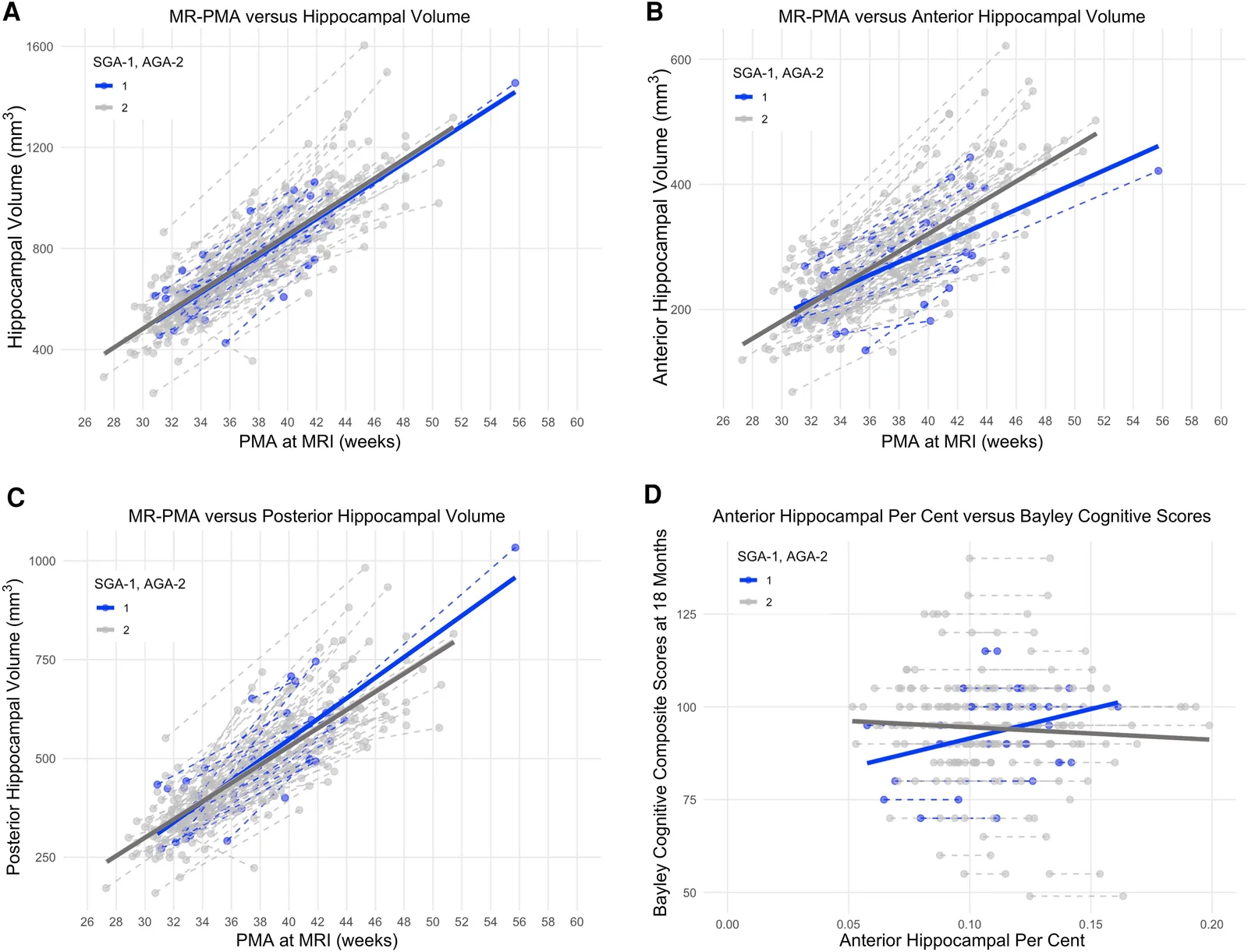
**Human hippocampal volume analysis.** (**A**) Total hippocampal volume and post-menstrual age (PMA) at MRI. (**B**) Anterior hippocampal volume and PMA at MRI. (**C**) Posterior hippocampal volume and PMA at MRI. (**D**) Neonatal anterior hippocampal percentage and Bayley Cognitive Composite scores at 18 months. Small for gestational age (SGA) are shown in blue and combined appropriate or large for gestational age (AGA) in grey. Volumes (graphs in **A**, **B** and **C**) and percentages (graph in **D**) measured at early life and term-equivalent age. Lines between points indicate growth trajectory between two measurements.

**Table 2:** Neonatal characteristics of human preterm infants in small for gestational age (SGA) group and appropriate for gestational age (AGA) group

	SGA (*n* = 20)	AGA (*n* = 139)	*P*
Birthweight, g; median [IQR]	690 [640–945]	940 [715–1225]	
Male, *n* (%)	10 (50%)	79 (55.8%)	0.74
Birth gestational age, weeks; median [IQR]	28.7 [26.8–29.9]	26.9 [25–28.9]	**0**.**02**
Need resuscitation at birth, *n* (%)	20 (100%)	137 (99%)	0.99
Antenatal steroids, *n* (%)	18 (90%)	113 (82%)	0.60
Apgar score < 8 at 5 min, *n* (%)	5 (25%)	72 (54%)	**0**.**03**
Hypotension requiring treatment, *n* (%)	6 (30%)	47 (34%)	0.90
Chronic lung disease, *n* (%)	3 (15%)	31 (22%)	0.65
NEC stage 2 or higher, *n* (%)	3 (15%)	18 (13%)	0.99
ROP requiring treatment, *n* (%)	0 (0%)	10 (7%)	0.46
PDA requiring treatment, *n* (%)	2 (10%)	39 (28%)	0.14
Major surgery, *n* (%)	2 (10%)	28 (20%)	0.44
Multiple infections (≥3 infection), *n* (%)	2 (10%)	15 (11%)	0.99
Days on mechanical ventilator, median [IQR]	1 [0–8]	6 [1–29]	**0**.**01**
Postnatal corticosteroids, *n* (%)	1 (5%)	41 (31%)	**0**.**04**
Morphine exposure, *n* (%)	6 (32%)	42 (32%)	0.99
Midazolam exposure, *n* (%)	1 (5%)	16 (12%)	0.61
Morphine and/or midazolam exposure, *n* (%)	6 (32%)	45 (34%)	0.90
PMA at early-life, weeks; median [IQR]	33.1 [32.4–34.2]	32.7 [31.7–34.3]	0.30
PMA at term-equivalent age, weeks; median [IQR]	41.6 [40.1–42.9]	41.2 [39.3–44.2]	0.96
WMI volume > 40 mm^3^ at early-life, *n* (%)	0 (0%)	15 (12%)	0.24
IVH grade 3 or 4 at early-life, *n* (%)	0 (0%)	18 (14%)	0.17
Hippocampal volume at early-life, mm^3^; median [IQR]	602 [532–640]	582 [523–657]	0.76
Hippocampal volume at term-equivalent age, mm^3^; median [IQR]	907 [769–1009]	906 [793–1033]	0.80
TCV at early-life, ml; median [IQR]	187 [158–206]	186 [165–208]	0.69
TCV at term-equivalent age, ml; median [IQR]	356 [315–371]	370 [308–409]	0.19
Bayley Cognitive Composite Score at 18 months CA, median [IQR]	95 [88–100]	95 [90–100]	0.68

Data are presented as *n* (%) or median [interquartile range, IQR]. CA = corrected age; IVH = intraventricular haemorrhage; NEC = necrotizing enterocolitis; PMA = post-menstrual age; ROP = retinopathy of prematurity; PDA = patent ductus arteriosus; TCV = total cerebral volume; WMI = white matter injury.

## Discussion

This study demonstrates that infants born FGR or SGA exhibit early life hippocampal structural deficits, mediated by reduced dendritogenesis of pyramidal neurons during fetal development, which are sustained and indeed more pronounced into infancy. The adverse intrauterine environment mediated by placental insufficiency limits fetal growth and induces a proinflammatory and pro-oxidative stress response in the FGR newborn lambs. After birth, despite adequate weight gain in infancy and normalization of the pro-oxidant and inflammatory milieu, hippocampal deficits in FGR lambs remained evident and significantly worsened after birth. This is the first cellular evidence for reduced hippocampal dendritogenesis *in utero* in FGR offspring that programmes sustained adverse hippocampal development after birth. Similar results were observed in our human cohort, demonstrating a reduced trajectory of early life postnatal anterior hippocampal volume growth in very preterm SGA infants compared to AGA very preterm infants, with subsequent consequences for cognitive function at toddler age.

The most striking finding of the current study is the novel identification that in both clinical and preclinical cohorts, FGR/SGA was associated with reduced trajectory of hippocampal growth (area or volume, respectively) in early life. Morphological analysis of individual CA1 neurons from the sheep ventral hippocampus (corresponding to the anterior hippocampus in humans) revealed that impaired hippocampal area is driven by disrupted dendrite outgrowth in the early postnatal period. At 4 weeks of age, but not newborn age, total ventral hippocampal area was reduced in FGR lambs, underpinned by a lack of expansion of the SR and SL-M hippocampal subregions, corresponding to the apical dendrite area. These neuropathology findings in the hippocampus were not caused by neuronal loss. Disruption to dendritogenesis of individual pyramidal neurons, and reduced hippocampal area growth, establishes the cellular basis for the MRI results observed in SGA human infants. The results in the clinical cohort both confirm and provide additive novel insight that hippocampal volume deficits apparent in SGA newborns are mediated, at least in part, by altered neuronal morphology, and critically link the programming of reduced dendritogenesis caused by *in utero* compromise with long-term cognitive dysfunction. Even when considering FGR lambs and SGA very preterm neonates, the translational implications of these findings are significant, demonstrating that meaningful improvements in learning and behaviour that are mediated by the hippocampus must target antenatal dendritogenesis.

In our human very preterm infants born SGA, we found a significantly reduced trajectory of anterior hippocampal volume expansion in the first weeks after preterm birth. The use of two MRIs, the first soon after very preterm birth and the second at term-equivalent age, permitted mapping of normal hippocampal growth in AGA very preterm babies, and reduced growth in the anterior hippocampus of SGA babies. Total hippocampal volume was not affected, but rather reduced growth rate was confined to the more stress-sensitive anterior hippocampus.^24^ Differences in hippocampal subregion development have been detected previously including in the growth rate of the anterior and posterior hippocampus,^37^ and in response to perinatal compromise,^24,38^ with the anterior hippocampus more vulnerable to developmental stressors.^24^ The functional consequences of deficits specific to the anterior hippocampus are unknown, but others have described that hippocampal lateralization and subregion maldevelopment mediate distinct contributions to memory formation.^18,39-42^ Of note is that all babies in the clinical study were born very preterm, with SGA infants having older gestational age at birth, less postnatal corticosteroid exposure, better Apgar scores at 5 min and shorter days of mechanical ventilation than AGA infants. Clinical characteristics, including midazolam exposure, pain, and severe intraventricular haemorrhage (IVH), which have been previously associated with hippocampal development in preterm infants, were comparable between SGA and AGA very preterm infants in this cohort. Importantly, the distribution of clinical factors previously linked with impaired hippocampal growth strengthens the conclusion that SGA is independently associated with hippocampal development in very preterm infants.

Children who are born FGR/SGA are at risk of suboptimal neurodevelopmental outcomes, including attention dysfunction in the neonatal period,^17^ and learning and memory deficits from two years of age^43-45^ persisting to school age^45,46^ and beyond.^47,48^ Here, FGR lambs displayed impaired cognition and memory, making more errors than appropriately grown lambs in the T-maze task. Functional assessments in lambs are limited, with the T-maze first described in 1968 to assess the senses used by ewes to detect their lambs^49^ and subsequently adapted to test memory and learning in young lambs.^29^ FGR lambs in the current study made more errors in the maze, but with a reduction in the time taken to complete the maze over successive weeks. This result suggests reduced self-regulation and impulsive decision-making, traits that are frequently observed in children born FGR/SGA as deficits in attention, executive function and behavioural control.^18,50-52^ Thus, the altered behavioural phenotype observed in our FGR lambs may represent an early neurobiological signature of later-emerging cognitive and behavioural difficulties. Critically in our clinical cohort, SGA infants displayed a significant correlation between anterior hippocampus volume and Bayley Cognitive Composite scores at 18 months’ corrected age, validating the hippocampal structure–function relationship.

Dendritogenesis and neuronal connections are driven by novel experiences and brain growth factors, particularly brain-derived growth factor (BDNF).^3,18,21^ We have previously shown that hippocampal BDNF expression is significantly reduced in the brain of FGR fetal sheep during late gestation.^25^ After birth, the lambs in this study were exposed to the same postnatal environment and both groups gained weight across a similar trajectory. Thus, differences observed in neuronal morphology were due to a suboptimal *in utero* environment, which programmed for continued aberrant postnatal neurodevelopment. In the newborn cohort of FGR lambs at 24 h after birth we detected elevated circulating cytokine IL-6 concentration and increased hippocampal microglia with an amoeboid phenotype, which together indicate a proinflammatory state caused by placental insufficiency. *In vitro* primary mixed neuronal culture studies have shown that proinflammatory cytokines directly reduce the number of primary dendrites, length and complexity,^53,54^ while *in vivo* fetal sheep work demonstrates that neuroinflammation is associated with reduced dendrite length and arborization.^55^ These findings provide insight that placental insufficiency induces a fetal systemic proinflammatory and oxidative stress response, neuroinflammation and reduction in brain growth factors that all likely contribute to the pathogenesis of altered neurite outgrowth of hippocampal neurons during fetal development. In turn, this adverse antenatal environment elicits a programming effect for poor postnatal neuron development and connectivity.

Very preterm infants display a complex constellation of motor and cognitive disabilities that are not fully explained by necrotic brain injuries such as periventricular leukomalacia or punctate white matter injury.^56^ These neurodevelopmental impairments are increasingly linked to dysmaturation of the cerebral grey matter and white matter with widespread cellular maturational disturbances linked to specific clinical exposures, including growth^57^ and nutrition.^58^ In very preterm infants, the hippocampus undergoes a robust period of *ex-utero* growth equivalent to the third trimester of gestation.^36^ Relative to full-term infants, very preterm infants with perinatal risk factors have reduced hippocampal volumes at term-equivalent age, associated with lower cognitive development at 2 years of age.^59^ Consistent with our serial imaging findings in very preterm SGA infants, another study showed reduced hippocampal volume at a single time point in preterm infants with FGR compared to age-matched peers.^17^ In findings extending neonatal brain imaging at term-equivalent age to 7 years of age, hippocampal growth between infancy and 7 years was less in very preterm children compared with the full-term group.^46^ These findings reinforce the long-term consequences of altered perinatal brain development. In infants born very preterm, stressors encountered in the postnatal environment are independent contributors to reduced hippocampal volume, including neonatal clinical care factors such as invasive procedures, days on ventilation and lower gestational age.^60^ In the current clinical study, all infants were born very preterm, and those born SGA were of slightly older gestational age.

We acknowledge the limitations of this study. First and importantly, we acknowledge that although the preclinical and clinical studies are complementary, they are subtly different. FGR in fetal sheep was induced via a surgical technique that causes placental insufficiency^26^ and lambs were born late-preterm/near-term, whereas the human infants were diagnosed with SGA based on birth weight only, and all human infants were born very preterm. In very preterm human infants, we used SGA as our clinical marker of poor fetal growth, defined by birthweight less than the 10th percentile on the Fenton Growth Chart.^35^ SGA is routinely used to describe high-risk small babies, particularly for neonatal studies in the absence of antenatal Dopplers to show placental dysfunction, as in our cohort.^14,61^ Importantly, both FGR and SGA infants have significantly elevated risks for perinatal morbidity and mortality and long-term neurological dysfunctions compared to infants who are appropriately grown, but we appreciate that these are worse in cases of FGR with a positive identification of placental dysfunction.^14,17,18,22-24,62^ All infants in the human study were born very preterm, which allowed a comparison of SGA versus AGA, but very preterm birth *per se* is a complication that adversely affects hippocampal growth^59^ and therefore future studies in the ovine group should specifically examine effects and factors that reduce neurodevelopment in FGR versus preterm cohorts. In the preclinical work, we were not powered to assess sex differences, and appreciate that there are altered functional outcomes associated with FGR that have different clinical presentation in males and females.^10^ The clinical cohort accounts for sex as a potential confounder; relative differences in anterior hippocampal size were not observed in males and females. We must also consider the application of the Golgi–Cox stain, which only stains ∼3% of neurons,^28^ and it is not known whether this selectivity is universal across morphologically normal and abnormal neurons. This low proportion of neuronal staining was, however, an advantage in the current study, particularly for the large dendritic arbours in the 4-week lamb cohort, in which complete dendrite length and branching could still be visualized within the CA1 hippocampal region without overlap from neighbouring neurons. Furthermore, we acknowledge that inflammatory markers used within this study (Iba-1 and IL-6) were limited and do not provide a comprehensive investigation of the inflammatory profile, as Iba-1 is not specific for microglia, and IL-6 is a pleiotropic cytokine with pro- and anti-inflammatory effects.^63^ Finally, in the human SGA study, MRI measured total, anterior and posterior volume, with only anterior hippocampal growth demonstrating a difference. We did not perform differential comparisons of hippocampal regions in the preclinical sheep study, with the left ventral region collected for Golgi–Cox staining and analysis of CA1 neuron morphology and the corresponding right ventral region used for immunohistochemical staining. Hippocampal shape and form varies slightly across species,^23^ and as best as possible, we utilized the ventral region of the sheep hippocampus as a comparable match for the anterior hippocampus in the human newborn brain.^23^ The results obtained in our human cohort support that there are intra-hippocampal region-specific dendritogenic differences in growth and neuropathology, which were not identifiable across hippocampal regions in the developing sheep brain.

This study has demonstrated that FGR *in utero* disrupts the profile of dendritogenesis in individual hippocampal pyramidal neurons, with ongoing significant consequences for continued dendrite outgrowth, complexity, connectivity and function after birth. The inclusion of complementary preclinical and clinical data in this study has provided a unique opportunity to characterize the cellular-level neuronal morphological deficits that underpin MRI volume deficits in FGR/SGA infants. Very preterm SGA infants included in this study showed reduced anterior hippocampal volumetric growth between birth and term-equivalent age relative to AGA infants. Similarly, FGR lambs showed reduced hippocampal area growth after birth, underpinned by a reduced trajectory of hippocampal CA1 neuron dendrite lengthening and branching. In control lambs, we observed maturational increases between newborn age and 4 weeks of age in apical dendrite branching and length, and complexity of basal and apical arbours. These trajectories for increasing dendrite growth did not occur for any measure of neuronal morphology in FGR lambs. Other studies in fetal sheep,^64^ guinea pigs^65^ and rodents^66^ have reported single time-point deficits in hippocampal dendrites caused by hypoxia, with current results establishing that morphological deficits are sustained and worsen after birth, particularly for the longer apical dendrites that project to the deeper hippocampal layers. These findings support that disturbed dendritic outgrowth of hippocampal neurons in the fetal period mediate a persistent programming effect for postnatal brain growth and reduced brain volume. Anterior hippocampal volume deficits in the SGA newborns were correlated with lower Bayley Cognitive Composite scores, linking the programming of reduced dendritogenesis caused by *in utero* compromise with long-term cognitive dysfunction. The contributions of brain inflammation, oxidative stress and reduced BDNF towards the inhibition of dendrite outgrowth in both the antenatal and postnatal period provide a basis for future studies to explore neuroprotective therapies.

## Supplementary Material

awag061_Supplementary_Data

## Data Availability

Data are available upon request.
