# Supplementary material for: Hippocampal growth and function are reduced in the newborn following fetal growth restriction
Source: Brain. 2026 Feb 20;149(10):3538–51. doi: 10.1093/brain/awag061 (PMC13634647; doi:10.1093/brain/awag061)
Supplement: Supplementary file 1 [file awag061_supplementary_data.pdf]

Supplementary Table 1 Immunohistochemical methodology (antigen retrieval, protein block, primary and secondary antibodies).

	Antigen retrieval	Protein block	Primary antibody	Secondary antibody
**Astrocytes (Glial fibrillary acidic protein, GFAP)**	0.01 M citric acid buffer (pH 6)	5% NGS 30 min at RT	Mouse anti-GFAP (Sigma-Aldrich, #G3893) in 0.3% Triton-X PBS 1:400 dilution Overnight at 4°C	Goat anti-mouse in PBS 1:200 dilution 60 min at RT
**Microglia, (Ionized calcium binding adaptor molecule, Iba-1)**	0.01M citric acid buffer (pH 6)	10% NGS 30min at RT	Rabbit anti-Iba-1 (Fujifilm Wako, #019-19741) in 0.2% Triton-X PBS 1:1000 dilution Overnight at 4°C	Goat anti-rabbit in PBS 1:200 dilution 60min at RT
**Microtubule-associated protein 2 (MAP2)**	Citrate antigen retrieval buffer (10mM citrate, 0.05% Tween 20, pH 6)	10% NGS in 0.1% Triton-X PBS 45min at RT	Mouse anti-MAP2 (Thermo Fisher Scientific #MA1-19166) in 5% NGS and 0.1% Triton-X PBS 1:200 dilution Overnight at 4°C	Alexa fluor™ 594 goat anti-mouse in 5% NGS and 0.1% Triton-X PBS 1:300 dilution 90min at RT
**Neuronal nuclei (NeuN)**	0.01M citric acid buffer (pH 6)	5% NGS and 1% BSA 30min at RT	Mouse anti-NeuN (Chemicon International, #MAB377) in 0.3% Triton-X PBS and 1% BSA 1:200 dilution Overnight at 4°C	Goat anti-mouse in 0.1% Tween PBS 1:200 dilution 30min at RT
**Synapsin (Syp-1)**	Citrate antigen retrieval buffer (10mM citrate, 0.05% Tween 20, pH 6)	10% NGS in 0.1% Triton-X PBS 45min at RT	Rabbit anti-Syp-1 (Synapsin I, Abcam; #ab254349) in 5% NGS and 0.1% Triton-X PBS 1:200 dilution Overnight at 4°C	Alexa fluor™ 488 goat anti-rabbit in 5% NGS and 0.1% Triton-X PBS 1:300 dilution 90min at RT

BSA = Bovine Serum Albumin, **NGS =** Normal Goat Serum, **PBS =** Phosphate-Buffered Saline, **RT =** Room Temperature

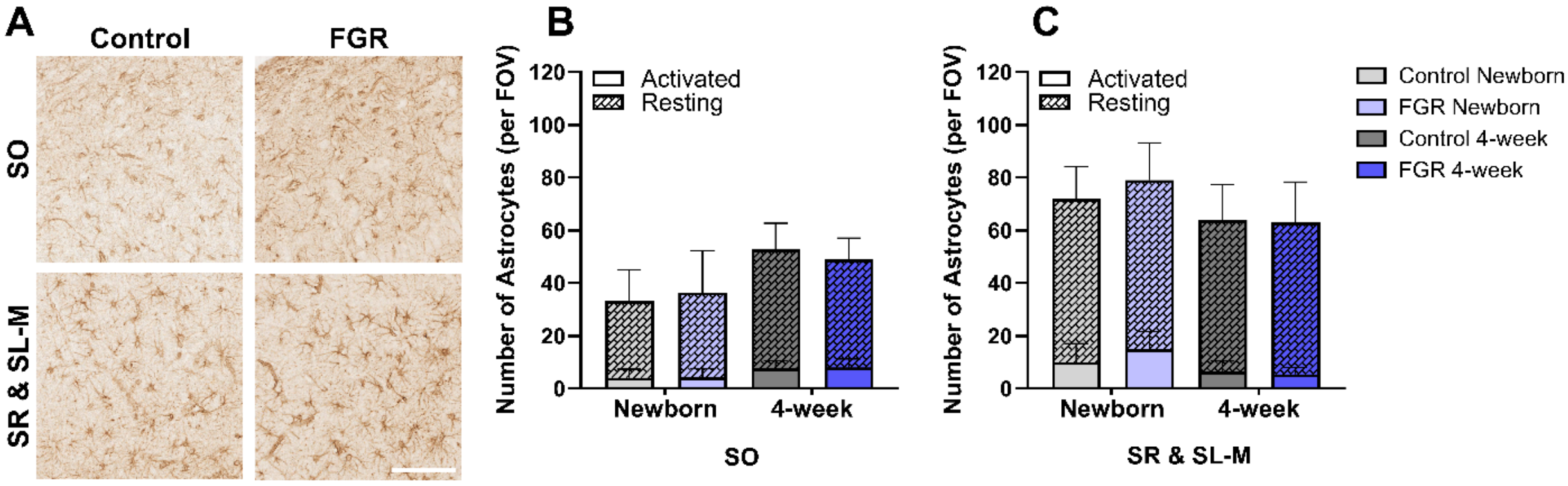

Supplementary Figure 1. Astrocyte immunohistochemical analysis within the hippocampus. Representative images of glial fibrillary acidic protein (GFAP) staining in newborn control and FGR lambs (A). GFAP positive cells quantified in morphological subtypes of activated and resting in Stratum Oriens (SO, B), and Stratum Radiatum and Stratum Lacunosum Moleculare (SR & SL-M, C) layers. Data are mean ± SD. Two-way ANOVA with age and group, bolded significant results, with Tukey's post-hoc test. $^{*}p<0.05$, $^{**}p<0.01$. Scale bar =1mm and 100μm. FOV = field of view.
